# Additive and Substractive Surface Structuring by Femtosecond Laser Induced Material Ejection and Redistribution

**DOI:** 10.3390/ma11122456

**Published:** 2018-12-04

**Authors:** Xxx Sedao, Matthieu Lenci, Anton Rudenko, Alina Pascale-Hamri, Jean-Philippe Colombier, Cyril Mauclair

**Affiliations:** 1 Laboratoire Hubert Curien, UMR 5516 CNRS, Université de Lyon, Université Jean Monnet, 42000 Saint-Etienne, France; anton.rudenko@univ-st-etienne.fr (A.R.); jean.philippe.colombier@univ-st-etienne.fr (J.-P.C.); cyril.mauclair@univ-st-etienne.fr (C.M.); 2 GIE Manutech-USD, 20 rue Benoit Lauras, 42000 Saint-Etienne, France; alina.hamri@manutech-usd.fr; 3 Mines Saint-Etienne, Univ Lyon, CNRS, UMR 5307 LGF, Centre SMS, F - 42023 Saint-Etienne, France; lenci@emse.fr

**Keywords:** ultrafast laser, femtosecond, ablation, scanning, additive surface structuring, hydrophobicity

## Abstract

A novel additive surface structuring process is devised, which involves localized, intense femtosecond laser irradiation. The irradiation induces a phase explosion of the material being irradiated, and a subsequent ejection of the ablative species that are used as additive building blocks. The ejected species are deposited and accumulated in the vicinity of the ablation site. This redistribution of the material can be repeated and controlled by raster scanning and multiple pulse irradiation. The deposition and accumulation cause the formation of µm-scale three-dimensional structures that surpass the initial surface level. The above-mentioned ablation, deposition, and accumulation all together constitute the proposed additive surface structuring process. In addition, the geometry of the three-dimensional structures can be further modified, if desirable, by a subsequent substractive ablation process. Microstructural analysis reveals a quasi-seamless conjugation between the surface where the structures grow and the structures additively grown by this method, and hence indicates the mechanic robustness of these structures. As a proof of concept, a sub-mm sized re-entrant structure and pillars are fabricated on aluminum substrate by this method. Single units as well as arrayed structures with arbitrary pattern lattice geometry are easily implemented in this additive surface structuring scheme. Engineered surface with desired functionalities can be realized by using this means, i.e., a surface with arrayed pillars being rendered with superhydrophobicity.

## 1. Introduction

Laser based additive manufacturing (AM) processes offer unprecedented new opportunities in design and manufacture [1]. In the vast majority of laser additive manufacturing (LAM) processes, high power, continuous, or long-pulsed lasers are commonly used as high-efficiency thermal sources to heat up metallic powders/bulk, and cause melting/cohesion. Ultrafast laser pulses, due to their low onset of thermal effect, on the one hand, are extensively used in the high-precision substractive processing sector, such as surface structuring [2,3], bulk inscription [4,5], and thin-film scribing [6]; however, on the other hand, they find themselves available for a fairly limited amount of applications in LAM. The few existing applications include ultrafast laser additive manufacturing/surface structuring for high melting temperature metals [7,8], laser-induced forward transfer (LIFT) for stacking up miniature building blocks together toward high precision, µm-scale additive processes [9,10], and improving surface finishing of metal parts produced by conventional LAM [11,12]. In this communication, we would like to broaden the spectrum of ultrafast LAM by exploring a concept of ultrafast laser additive surface structuring (LASS): by taking advantage of the material spallation/phase explosion induced by intense ultrafast laser irradiation, the energetically ejected material from the phase explosion can be transferred and accumulated to form a predefined structure. This approach is powder-free, and the structure building material is directly acquired from the substrate where the additive features are to be grown. Moreover, “finishing touches” can be made to the as-built structures by using the very same ultrafast laser, in order to improve the fineness of the surface finishing of the additively grown features. In the following, the concept is explained firstly. Then, as a proof of concept, two types of structures are fabricated using the proposed ultrafast LASS approach. The aspect of added functions at the surface with these ultrafast laser grown structures is also discussed.

## 2. Materials and Methods

**Figure 1:**
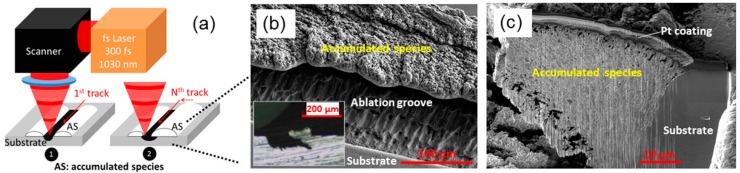
Ultrafast laser additive surface structuring (LASS) explained: (**a**) Laser scan path is indicated by the red arrows: the laser beam movement starts from the far side and advances linearly toward the near side to form a scan track. The scan tracks start from the right side and end on the left. The numbers 1 and 2 represent the beginning and the end of the process, respectively; (**b**) scanning electron microscope (SEM) micrograph showing the site of ablation, and additively grown part. A mechanically prepared cross-section is given as inset. (**c**) A Focused Ion Beam (FIB) milled cross-section of the additively fabricated part. The platinum layer appearing in the micrograph is a protective coating deposited prior to the milling process.

A schematic drawing of the laser setup and raster scan strategy is depicted in Figure 1a. For the sake of clarity, the material reported in the Results section is aluminum. In order to verify whether the proposed ultrafast LASS is generic, a few other materials were also chosen for the same test, such as copper and titanium alloy Ti6Al4V, and comparable results were obtained from these materials, too. Nonetheless, a similar test on stainless steel 316L did not yield satisfactory results, suggesting a certain material dependency and limitation of this process. The targets were prepared by conventional metallography procedures with a surface roughness Ra = 20 nm. The micromachining was carried out using a fiber fs laser system (Tangerine HP, Amplitude Systems, Pessac, France). The laser has a central wavelength of 1030 nm with a pulse duration of 300 fs, and a tunable repetition rate from single shot to two MHz. The linearly polarized laser pulses were attenuated, sent through a Galvano scanner, and focused through a 100-mm telecentric f-theta lens. The focused laser spot exhibits a Gaussian profile and the spot diameter (at 1/e^2^) measures 2*ω*_0_ = 22 µm. A raster scanning strategy was used. The overlap ratio of 0.91 between successive laser pulses and successive scan tracks was kept constant for all of the experiments. This relatively high overlap ratio was intended for a homogeneous energy deposition [13]. The laser fluence quoted in this paper is the peak fluence F = 2*ε*/π *ω*_0_^2^, with *ε* being the laser pulse energy.

The topographical analysis was performed using an optical microscope (OM, Zeiss, Oberkochen, Germany) and scanning electron microscope (SEM, FEI Europe B.V., Eindhoven, Netherlands). Cross-section samples for OM were prepared by wheel-saw halving, cold-setting resin mounting, and mechanical polishing. Cross-section samples for SEM inspection were made using a dual beam system SEM and Focused Ion Beam (FIB, FEI Helios Nanolab 600i dualbeam workstation). Energy-dispersive X-ray spectroscopy (EDX, Brucker, Bremen, Germany) was used to evaluate the surface chemistry change. A Vikers microindentation (Cetim, Saint-Etienne, France) was also performed to access hardness, with a loading force of two N. All of the hardness values quoted in the text were averaged values from five individual measurements. For the wetting test, a three-µL water droplet was deposited on the surface by a microsyringe, and the side-on view was captured by a dedicated camera. The contact angle was deduced from the registered images using software Digidrop (version 13.06.3.12GB, GBX, Romans sur Isère, France).

## 3. Results

The lower-right part of the schematic drawing shown in Figure 1a can be used to illustrate the concept of the LASS by intense ultrafast laser irradiation (roughly defined as fluence >5~10 times the ablation threshold). Then, the material experiences a photo excitation, electron–phonon non-equilibration-enhanced electron heat conduction before equilibrium with the lattice, and then undergoes spallation and phase explosion [14]. Various species, depending on the laser fluence, consisting of anything from liquid layers and/or large droplets to a mixture of vapor-phase atom clusters and droplets etc., are ejected as a consequence of stress release. Under certain circumstances (a more dedicated discussion is in the following paragraphs), the ejected species land on the adjacent site, adhere to the vicinity of ablation site, and form an accumulation. Such a material redistribution is somehow similar to the work of Temmler, et al. [15] (although the mechanism governing the material displacement is totally different). By raster scanning a small area on a substrate, the accumulation continues on and eventually forms a three-dimensional structure by itself, which will be termed as additive structures (AS), as indicated by the white bump in Figure 1a. An AS on aluminum sample was fabricated in this way, and its SEM micrograph is given in Figure 1b. The material to form the AS was sourced from the ablation groove, as marked in Figure 1b, at a laser fluence of 18 J/cm^2^, and 500-kHz repetition rate. The lateral dimension of the AS is about half of the groove width, and the longitudinal dimension is approximately 50 µm, which is better seen from the mechanical cross-section presented in the inset.

In order to characterize the AS, we performed microstructural and chemical analysis. The SEM micrograph in Figure 1c shows a FIB prepared cross-section from the substrate–AS junction region. Laminated structures are observed on the AS, the formation of which is due to ablative and accumulative processes. The cross-section elaborates also that the cohesion between the AS and substrate is at the microstructural level. A borderline is evident at the junction, but no major voids nor pores are located there, which implies a robust conjugation between the substrate and the AS. The AS is relatively dense. There are some sub-µm sized voids in the bulk region of the AS, but they are sparse. Nonetheless, it is evident that some micropores are located at the outermost surface region and the lower outer surface region. Those micropores located in the outermost surface region could be potentially eliminated by a substractive ablation process step, which is discussed later in this communication. An EDX spectrum was acquired from different locations across the entire AS, and compared with the spectrum obtained from the substrate. In the AS, a small increase of oxygen and nitrogen is deduced from their characteristic peaks’ intensity in the spectra. This chemical composition change may be due to an oxidation/nitration or air trapping (due to the presence of the sub-µm voids) within the AS during the process.

**Figure 2:**
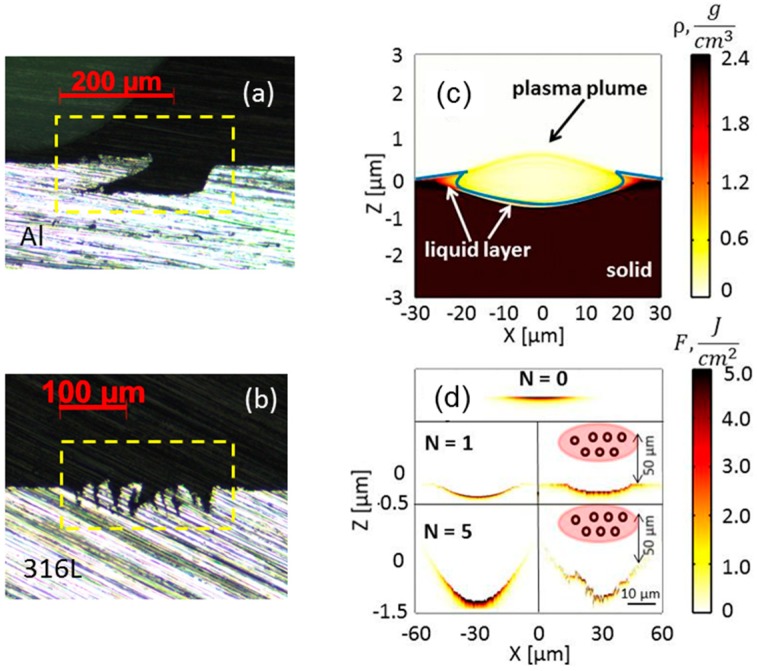
Cross-section images of laser processed (**a**) aluminum, and (**b**) stainless steel 316L, at a fluence of 18 J/cm^2^ and pulse repetition rate of 250 kHz. The dashed line boxes are visual guides for highlighting the features inside the ablation grooves; (**c**) Density snapshot showing plasma plume expansion from aluminum surface 50 picoseconds after irradiation; (**d**) Multi-pulse simulations of energy deposition on stainless steel surface. Top row N = 0, initial surface; middle row N = one pulse; and bottom row N = five pulses. In the middle and bottom rows, unaffected (left) /affected (right) laser structuring by the presence of nanoparticles of r = 50 nm and concentration of C = 50 µm^−2^.

As mentioned in Section 2, the AS can be achieved on aluminum but not stainless steel; a typical comparison can be viewed in Figure 2a,b, with the cross-sections of these two materials treated at a fluence of 18 J/cm^2^ and a 250-kHz repetition rate. The laser processing is shown to be strongly material-dependent in the ~100 kHz repetition rate regime [16,17], resulting either in material accumulation on the side of the ablation groove in the aluminum case, or to significant roughness development in the case of stainless steel. The laser plasma hydrodynamics and ablation particles shielding may be taken account of in these observations.

The interaction of ultrafast laser at fluence exceeding the ablation threshold with metal targets is associated with a large variety of physical phenomena, taking place from femtosecond up to microsecond timescales [14,18]. At 250 kHz (a pulse-to-pulse interval of four microseconds), the pulse-by-pulse thermal accumulation alone does not play a major role, as the irradiated surfaces have enough time to cool down during the interval. Besides thermal accumulation, the laser ablation by high-intensity pulses is accompanied by plasma plume expansion away from the metal target [18]. Figure 2c depicts the initiation of ablation simulated by two-dimensional (2D) compressible Navier–Stokes equations [2,19], taking account of Gaussian pulse energy deposition on the surface, lattice heating, and hydrodynamic movement, which causes strong pressure gradients and material removal. The lowest density corresponds to hot laser-induced plasma, the highest density corresponds to unaffected ablation area, and the intermediate density corresponds to a liquid state. The central part of the plume leaves the surface and expands longitudinally above the surface; however, the corners of the plasma plume remain less hot, may re-solidify before the arrival of the next pulse, and then form a redistributed aluminum layer above the initial level. In this case, the consequent pulse-by-pulse ablation with a scan from left to right leads to a pronounced microstructure on the left side of the laser-processed area, as displayed in Figure 2a. Other possible mechanisms of material re-deposition include Marangoni melt flow and recoil vapor pressure [15,20]. However, these effects fail to explain why the accumulated material is observed above the ablated area, but not outside it.

Another characteristic feature of the ultrashort laser ablation of metal targets is the formation of nanoparticles of sizes up to r = 100 nm in plasma plume [21,22,23,24,25]. The species are known to be moving much slower than the plasma plume, with the velocities slightly exceeding 100 m/s [22,25]. The presence of slow moving nanoparticles generated by a laser pulse may influence the following laser pulse interaction with the surface. To investigate the effects of light absorption on the surface, with and without transmission through nanoparticles, we use an ablation model, including Maxwell equations [26] coupled with electron-ion heat transfer equations [19]. The spherical nanoparticles with a radius r = 50 nm and a concentration of C = 50 µm^−2^ are distributed randomly in an ellipsoid-centered region at distance of 50 µm above the initial surface level, with a radius Rz = 10 µm in the propagation direction, and Rx = 20 µm in the transverse direction. The nanoparticles are assumed to be created by the first laser pulse. The numerical results show the surface profile after N = one and N = five pulse irradiation with and without taking account of the energy loss in transmission through nanoparticles in Figure 2d. Although only a slight difference is seen after the first pulse irradiation, the inhomogeneous distribution of the energy due to the presence of nanoparticles seriously degrades the ablation quality after five consecutive pulses: the ablation profile is now asymmetric, with roughness features up to a few hundred nm. It is worth mentioning that owing to the low reflectivity of stainless steel (R = 0.56) compared to that of aluminum (R = 0.96), the greater transmission of laser pulse through steel nanoparticles would cause a stronger surface roughness. The effects of the interaction between the nanoparticles and laser-induced plasma created by the next pulse may as well lead to uncontrolled material deposition inside the crater. Both phenomena may have an impact on the quality and/or surface morphology [22]. The difference in ablated volume and depth is revealed by simulations, taking into account the presence of nanoparticles in Figure 2d already after five pulses of irradiation. Thermal effects might be at the origin of further ablation crater degradation, as the diffusivity of stainless steel $D=k_{i}/\rho C_{i}\approx 5\times 10^{-6}\;m^{2}/s$ ($k_{i}$ is the lattice thermal conductivity, $C_{i}$ is the lattice heat capacity, and ρ is the lattice density), which is six times lower than the diffusivity of aluminum $D\approx 3\times 10^{-5}\;m^{2}/s$. For aluminum, the material accumulates only at the corners of the laser-processed area, leaving the central part inside the ablation crater free from micro-debris [16], and facilitating the proposed technique of additive surface structuring.

**Figure 3:**
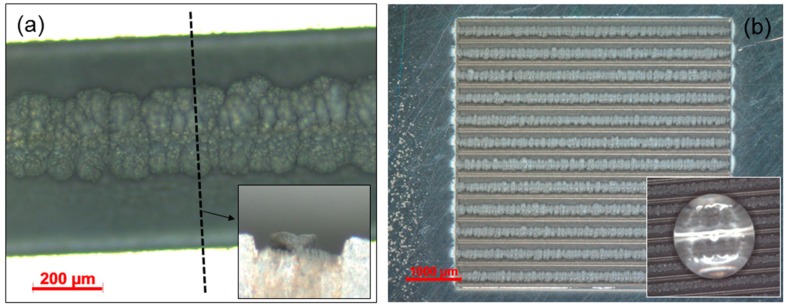
(**a**) Top view of the re-entrant structure realized by building the additive structures (AS) block from two sides. The dash line and the arrow are the visual indication of a cross-section. The inset is a cross-section of this re-entrant structure; (**b**) The structure unit shown in (**a**) is easily implemented on a large surface scale. This surface exhibits isotropic hydrophobicity (lower bottom inset).

The application of such AS for surface functionalization is multifold. One of the simplest is implemented by building two basic AS blocks against one to another, and is reported in Figure 3a. The inset is a mechanical cut cross-section revealing the negatively inclining feature of the sidewalls of the AS. Such geometry is otherwise termed the re-entrant shape, and is capable of rendering a surface liquid repellent property [27]. In order to test this functionality, a large surface of a few mm^2^ area was fabricated with repeating “re-entrant” pattern periodically (Figure 3b). The surface was hydrophilic and anisotropic right after the LASS process, but turned to isotropic and hydrophobic after a thermal treatment at 150 °C for two hours: a droplet could sit on the AS surface without spreading, as shown in the inset. Such surface morphology, once capped with a low surface energy coating, might also exhibit oleophobic characteristics [28]; experiments on coating preparation and oleophobicity test are currently underway. It is also worth noting that upscaling such ultrafast LASS to an even larger surface, in the order of cm^2^ towards m^2^, is fairly straightforward nowadays, as it is based on standard ultrafast laser beam shaping, scanning, and processing tools [3].

**Figure 4:**
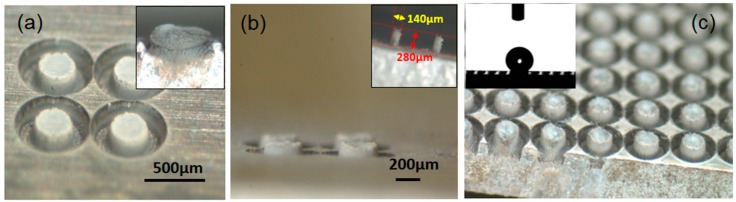
(**a**) Four sub-mm sized pillars made by the ultrafast laser additive surface structuring (LASS) process, plus ultrafast laser substractive ablation at the contour of the pillar blocks to sharpen the edges. The inset shows a single AS unit without its edge being sharpened; (**b**) Tilted view of the pillars. The pillars can be grown higher, at the cost of pillar diameter, as shown in the inset; (**c**) Pillars similar to those in (**a**) are fabricated on a large scale for achieving a superhydrophobicity at the surface, as demonstrated in the inset.

Furthermore, an additional merit offered by the ultrafast LASS process is worth illustrating. Given that the ultrafast laser is also a high-precision micromachining tool, additional laser processing steps can be added after the AS is fabricated, and the surface finishing of the AS can be improved by using the very same laser source. For instance, the edges of AS can be sharpened by ultrafast laser ablation, as shown in Figure 4. The four sub-mm sized pillars were fabricated by the LASS process (by circulating the laser beam from the center to outer region), and they had a mushroom-shaped geometry right after the process (a side-on view of the mushroom-shaped AS is given as an inset in Figure 4a). The cap part of the “mushroom” is trimmed to a pillar shape, as shown in Figure 4a, after an additional ultrafast laser substractive scan along the contour of the AS at a reduced laser fluence (7 J/cm^2^). The sharp edge is better viewed in Figure 4b, where the sample is tilted by a nearly 90° angle. The pillars in this particular case are 250 µm in diameter and 100-µm high. Pillars with height (the elevation from the substrate surface to the peak of the pillars) up to approximately 300 µm and small diameters can also be made by this strategy; the side-on view is given in the Figure 4b inset. The large surface production of the AS, which is similar to those in Figure 4a, is also easily implemented, and an example is presented in Figure 4c. The inset in Figure 4c is a wetting test on the surface with a large array of sub-mm sized pillars, where a superhydrophobicity contact angle of 150 °C is observed (after a thermal treatment at 150 °C for two hours). Mechanical property-wise, the microindentation test revealed a decreased hardness at the surface of the AS, a Vickers hardness value at two N load HV_2N_ = 230 at the AS, compared to a Vickers HV_2N_ = 252 of the substrate. The reduction of the hardness is thought to be related to the presence of the micropores and reduced density in the AS compared to bulk aluminum. It is obvious that the substractive process step can be applied to remove the previously mentioned micropores lodged in the outermost surface region. If this surface porous layer were removed, the microhardness would be further improved. A more comprehensive study on this matter and process optimization will surely be meaningful toward further exploring the value of this additive and subtractive surface structuring approach.

## 4. Discussion

This report proposes a novel ultrafast LASS approach that is based on intense femtosecond laser irradiation, ablation, and subsequent material ejection. The inhomogeneous distribution of hot plasma induced by intense laser irradiation contributes to the accumulation of ablated species near the ablation area. The accumulation leads to the formation of the additive manufactured structures. As a proof of concept, one-dimensional re-entrant surface structures are fabricated using the approach. The advantage of ultrafast laser refining the additively manufactured structures is also demonstrated. These femtosecond laser additively manufactured geometries, once fabricated on a large scale, can render the underlying surfaces with desired properties such as anisotropic wetting and superhydrophobicity. Although the above results were obtained from aluminum substrates, further study suggests that the method is quite generic and applicable to certain other metals and/or alloys. Supplementary tests have confirmed the applicability of this process mode to copper and titanium alloy Ti6Al4V, at adapted laser fluences. Nonetheless, a similar test on stainless steel 316L did not yield satisfactory results. Numerical analysis suggests that the presence of laser-induced nanoparticles, optical properties such as reflectance and transmission, and material properties such as diffusivity may play a role in the observed discrepancies in stainless steel with respect to the other metal/alloys. It is clear that a better understanding is still required toward the further development of the process. In the meantime, the mechanical strength of these structures needs to be characterized quantitatively. Surface functionalization, especially potentials in adding superomniphobic (hydrophobic and oleophobic at the same time) properties to an engineered surface, will be explored.
